# The Japanese Version of the Massachusetts General Hospital Acupuncture Sensation Scale: A Validation Study

**DOI:** 10.1155/2017/7093967

**Published:** 2017-06-06

**Authors:** Masako Nishiwaki, Miho Takayama, Hiroyoshi Yajima, Morihiro Nasu, Jian Kong, Nobuari Takakura

**Affiliations:** ^1^Department of Acupuncture and Moxibustion, Faculty of Health Sciences, Tokyo Ariake University of Medical and Health Sciences, 2-9-1 Ariake, Koto-ku, Tokyo 135-0063, Japan; ^2^Department of Physiology, Showa University School of Medicine, 1-5-8 Hatanodai, Shinagawa-ku, Tokyo, Japan; ^3^Department of Psychiatry, Massachusetts General Hospital, Harvard Medical School, Charlestown, MA 02129, USA

## Abstract

Acupuncture sensations are considered essential in producing the treatment effect of acupuncture. The Massachusetts General Hospital Acupuncture Sensation Scale (MASS) is a frequently used scale in acupuncture research to measure acupuncture sensations. We translated the MASS into Japanese (Japanese MASS) based on Beaton's guidelines. 30 acupuncturists evaluated the relevancy and meaning of the 12 descriptors included in the Japanese MASS. The content validity ratios for 10 of the 12 descriptors were 0.33 or greater. 42 healthy subjects then evaluated acupuncture sensations evoked by manual acupuncture at LI4 using the Japanese MASS. Cronbach's alpha was 0.86. The correlation coefficient of total MASS scores and total Short Form McGill Pain Questionnaire scores and MASS indices and sensory visual analogue scores were 0.78 and 0.80, respectively. Factor analysis loaded the 12 descriptors onto two meaningful factors. This study demonstrated that the Japanese MASS has good reliability, content validity, criterion-related validity, and construct validity. Therefore, the Japanese MASS is a valid and reliable instrument for use with Japanese populations.

## 1. Introduction

Acupuncture sensations, or de qi, felt by patients are sensations elicited with needling and are traditionally considered to play an important role in acupuncture treatment [1–3]. Characteristics of de qi elicited with needling depend on where and how a needle is applied and on the patient's state of health [4]. The Standards for Reporting Interventions in Clinical Trials of Acupuncture recommend that manuscripts related to clinical acupuncture trials report de qi, demonstrating its importance in acupuncture research [5].

Studies suggest that there are close associations between acupuncture sensations and acupoints [6–8], anatomical structures (e.g., the tissues and the nerves stimulated with needle insertion) [9, 10], depth of needle insertion [11], and methods of acupuncture [6, 12–14]. Physiological responses with de qi, including blood flow [15, 16] and brain activity (observed using electroencephalography [17] or functional magnetic resonance imaging [18–20]), have also been reported. Studies investigating physiological or psychological responses induced with acupuncture sensations, as well as those focused on the characteristics of acupuncture sensations per se using globally common questionnaires, are indispensable in evaluating the relationship between acupuncture sensations and the effects of acupuncture.

The Acupuncture Sensation Scale (ASS) [21], the Massachusetts General Hospital Acupuncture Sensation Scale (MASS) [22], and the Southampton Needle Sensation Questionnaire (SNSQ) [23] were developed to qualify and quantify acupuncture sensations and investigate their relationship to the effect of acupuncture. The development of the ASS was based on McGill's Pain Questionnaire (MPQ) [21], the MASS on traditional Chinese medicine descriptions of de qi sensation [2, 6, 22], and the SNSQ on interviews with patients [23]. They have been validated for use in clinical acupuncture trials and physiological studies.

In order to use these acupuncture sensation scales to investigate patients' acupuncture sensations in languages other than English, the scales must be translated into the respective language. The ASS, MASS, and SNSQ have been translated into Korean [24], Chinese [25], and German [26], respectively. Some of these translations have been used in acupuncture studies evaluating de qi [27, 28].

However, only the ASS has been translated into Japanese, with the descriptors adopted from the Japanese version of the MPQ [29]. Although the MPQ in Japanese was validated, the Japanese version of the ASS has not yet been validated. Therefore, researchers whose native language is Japanese are not able to conduct acupuncture trials with Japanese patients using a validated Japanese acupuncture sensation scale. As the MASS is the most well-known and frequently used scale, we decided to develop a validated MASS in Japanese rather than validating the ASS in Japanese.

In this study we aimed to develop a Japanese language version of the MASS, the English version of which is the most used sensation scale in acupuncture studies [2, 3, 18]. We translated the original MASS into Japanese (Japanese MASS) based on Beaton's guidelines and tested both its reliability and its validity.

## 2. Methods

This cross-cultural adaptation of the MASS from English to Japanese comprised translation (Beaton's stages 1–4) and validation and reliability tests (Beaton's stage 5) [30]. The study was approved by the Ethics Committee of Tokyo Ariake University of Medical and Health Sciences (TAU). The study objectives and protocol were fully explained to each participant using a written form, and all participants provided written consent.

### 2.1. Translation and Production of the Prefinal Version of the Japanese MASS

The original English version of the MASS was translated into Japanese according to a five-step process for cross-cultural adaptation, as described by Beaton et al. [30].

In the first stage, two acupuncture researchers whose native language was Japanese made forward translations from English to Japanese. In the second stage, the two independent translations made by the acupuncture researchers were synthesised into a Japanese version (synthesised version), based on a discussion between the two translators. In the third stage, two native-born Americans whose native language was English produced two English backtranslations of the synthesised version. One backtranslator was an acupuncturist. The other was not an acupuncturist and had no medical/acupuncture background and no prior knowledge of or information about the MASS. In the fourth stage, an expert committee comprised of three licensed acupuncturists (all of whom held doctoral degrees in medicine) from the TAU Department of Acupuncture and Moxibustion, along with a Boston-based Japanese acupuncturist who had obtained her acupuncture license in Massachusetts, reviewed the original MASS, the forward translations, the synthesised version, and the two backtranslations. They discussed the material for semantic, idiomatic, experimental, and conceptual equivalences between the original MASS and the synthesised version to develop a prefinal version of the Japanese MASS.

### 2.2. Content Validity

In the fifth stage, we recruited 30 licensed acupuncturists (19 males, 11 females) to evaluate the content validity of the Japanese MASS, based on Lawshe's approach [31]. The mean age (± standard deviation [SD]) of these acupuncturists was 41.0 ± 11.4 years and the mean experience in acupuncture treatment was 12.3 ± 8.7 years. We asked the acupuncturists to evaluate the necessity for each descriptor (heaviness, dull pain, tingling, warmth, sharp pain, numbness, throbbing, deep pressure, aching, soreness, cold, and fullness/distension) as an acupuncture sensation on a five-point scale: “agree,” “somewhat agree,” “neutral,” “somewhat disagree,” or “disagree.”

### 2.3. Internal Consistency, Criterion-Related Validity, and Construct Validity

We recruited 42 healthy subjects (20 males, 22 females, aged 21.2 ± 1.5 years) who had normal Japanese language ability and no nervous system disorders. Half of these 42 subjects had previous acupuncture experience and knowledge about acupuncture (including acupuncture sensations or de qi), whereas the others had no experience of acupuncture and no knowledge about acupuncture. The study was conducted in a laboratory at TAU, Tokyo, Japan. The acupuncture room was kept at 24–26°C. Acupuncture was administered by five acupuncturists with 14.8 ± 4.4 years of acupuncture experience.

Subjects lay on their back on a bed with their arms resting along their sides. An acupuncturist disinfected the subject's skin with ethanol, administered the needle using the tapping-in method to penetrate the skin, and then applied the rotation technique at LI4 (large-intestine meridian) [6, 7, 12, 14, 16, 21, 24, 25, 27, 29] on the right or left hand. After the needle was inserted 10 mm perpendicularly to the skin's surface, the acupuncturist rotated the needle handle by 180 degrees clockwise and anticlockwise at 1 Hz for 2 minutes [7] and then removed it. The needles were stainless steel (0.18 mm diameter, 40 mm length; Seirin, Shizuoka, Japan).

To exclude bias from acupuncture naïve or nonnaïve subjects, we randomly assigned acupuncture-experienced and non-acupuncture-experienced subjects to right LI4 or left LI4 groups using a web-generated table of random numbers (http://randomization.com).

Immediately after the removal of the needle, subjects completed three questionnaires on their acupuncture sensations during the needle rotation: (1) the sensory visual analogue scale (S-VAS), on which subjects rated the intensity of sensations from 0 (no sensation) to 100 (most severe sensation, unbearable); (2) the Japanese MASS (prefinal version), on which subjects rated the magnitude of each acupuncture sensation corresponding to the 12 descriptors on a numerical rating scale (NRS) from 0 (none) to 10 (strongest imaginable); if the subjects felt other sensations besides the 12 descriptors, they described the additional sensation and rated it on the NRS; (3) the Japanese version of the Short Form MPQ (SF-MPQ), which consists of the same sensory and affective descriptors as the original English version of the SF-MPQ. As the reliability of the Japanese SF-MPQ has been demonstrated, it may be used to evaluate criterion-related validity to show the concurrent validity of the Japanese MASS.

### 2.4. Statistical Analysis

To evaluate the need for each descriptor as an acupuncture sensation and the content validity of the Japanese MASS, we calculated the content validity ratio (CVR) from the scores of all 30 acupuncturists using the formula used by Lawshe [31]. A 5% significance level represents a CVR of 0.33 when there are 30 acupuncturists [31].

To estimate the reliability of the Japanese MASS, we calculated the Cronbach's alpha coefficient using the scores obtained from all subjects to assess internal consistency. To evaluate the criterion-related validity to show concurrent validity, we calculated Pearson's correlation coefficients between the total MASS scores and S-VAS scores, total SF-MPQ scores, and the sensory and affective dimension scores in SF-MPQ, respectively. We also calculated Pearson's correlation coefficients between the MASS indices and the same scores. The MASS index is an exponential smoothing average, which is considered a more reliable method to measure the magnitude of overall acupuncture sensations than total MASS scores. We calculated the MASS indices using scores for the 12 descriptors [22].

Based on the original MASS study [2], we conducted an exploratory factor analysis for the Japanese MASS to demonstrate construct validity. Factor extraction was performed using a principal component method, in which the number of factors was determined by scree plot criteria with eigenvalues. We conducted a varimax rotation involving Kaiser normalisation below 25 iterations for convergence.

Statistical analyses were performed with SPSS Version 24 (SPSS Japan Inc., IBM Company, Tokyo, Japan).

## 3. Results

### 3.1. Translation into Japanese

**Figure 1:**
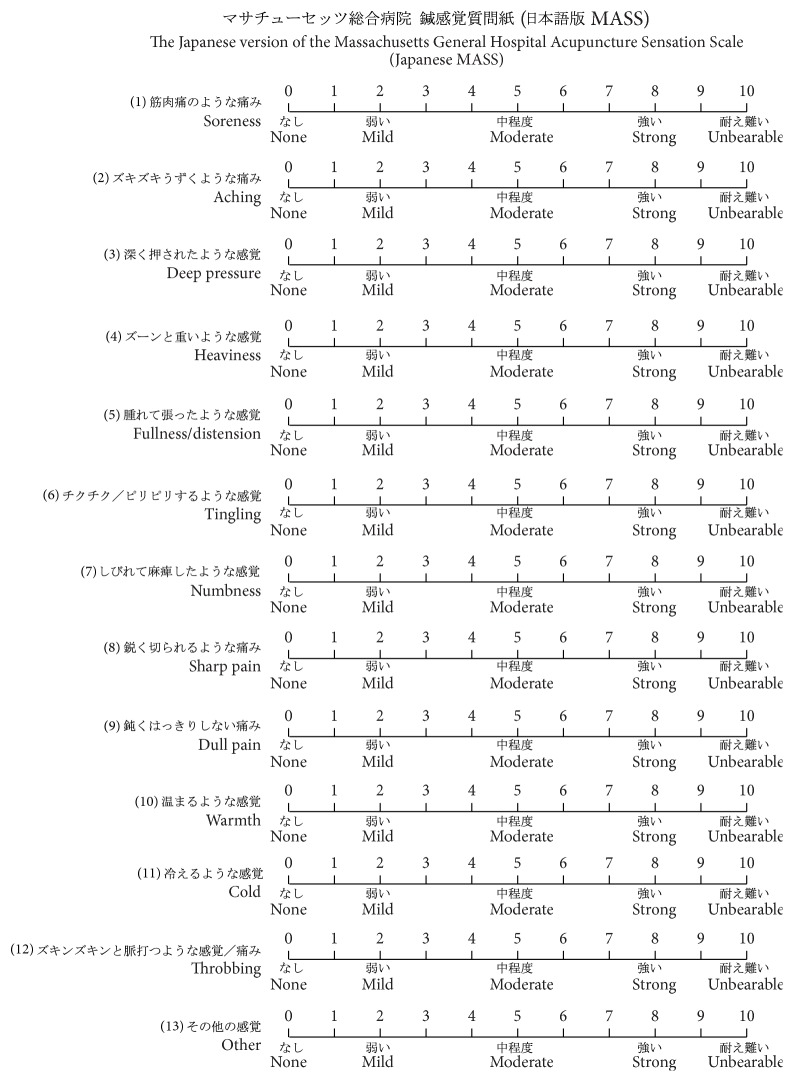
Japanese MASS. Each descriptor in English is as follows: (1) soreness, (2) aching, (3) deep pressure, (4) heaviness, (5) fullness/distension, (6) tingling, (7) numbness, (8) sharp pain, (9) dull pain, (10) warmth, (11) cold, (12) throbbing, and (13) other. Intensities under each scale in Japanese (in English) are as follows: 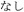 (none), 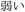 (mild), 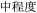 (moderate), 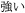 (strong), and 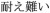 (unbearable).

Figure 1 shows the Japanese MASS. In the forward translation, “soreness” in the English MASS was translated as “feeling of languid” in Japanese. However, “feeling of languid” was backtranslated as “sluggish” in English. Therefore, the expert committee changed “feeling of languid” to “muscular pain” in Japanese, which was backtranslated as “soreness” in English. There were no revisions required for other descriptors during the translation process.

### 3.2. Content Validity

**Figure 2:**
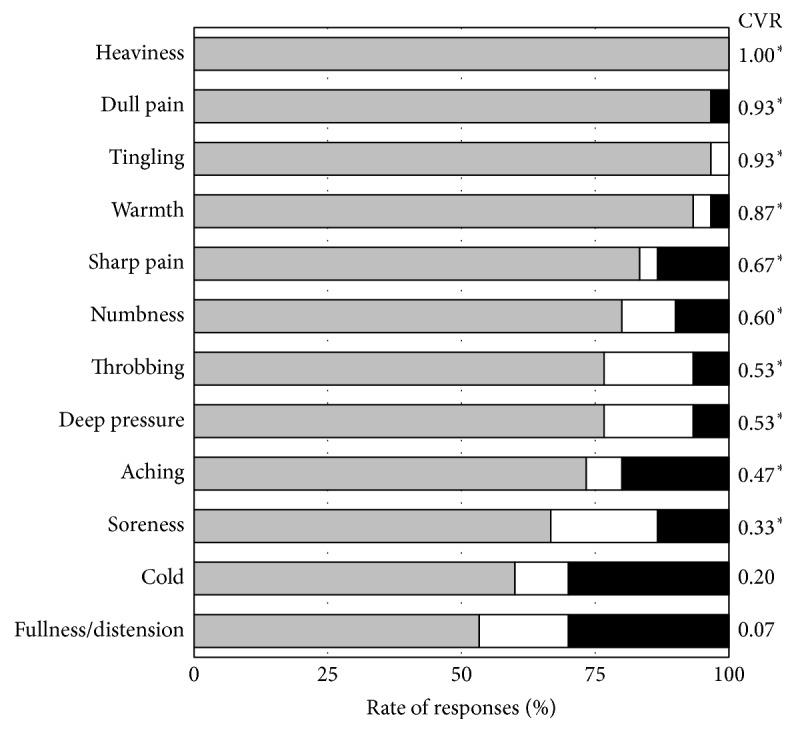
Rate of responses for each descriptor (acupuncture sensation) and content validity ratios (CVR, ^*∗*^*p* < 0.05) (*n* = 30). The grey bars indicate “agree” and “somewhat agree,” the white bars indicate “neutral” and the black bars indicate “somewhat disagree” and “disagree.”

Figure 2 shows the CVRs calculated from the data reported by the 30 acupuncturists for each descriptor in the Japanese MASS. Overall, the CVRs for the 12 descriptors ranged from 0.07 for “fullness/distension” to 1.00 for “heaviness.” We found significant validity for the 10 CVRs, with the exception of “fullness/distension” and “cold.” In particular, the descriptors “heaviness,” “tingling,” “dull pain,” and “warmth” which showed high validity.

### 3.3. S-VAS Scores, Total MASS Score, and MASS Index

The mean ± SD intensity of acupuncture sensations marked on the S-VAS by the 42 subjects was 38.8 ± 25.6.

**Figure 3:**
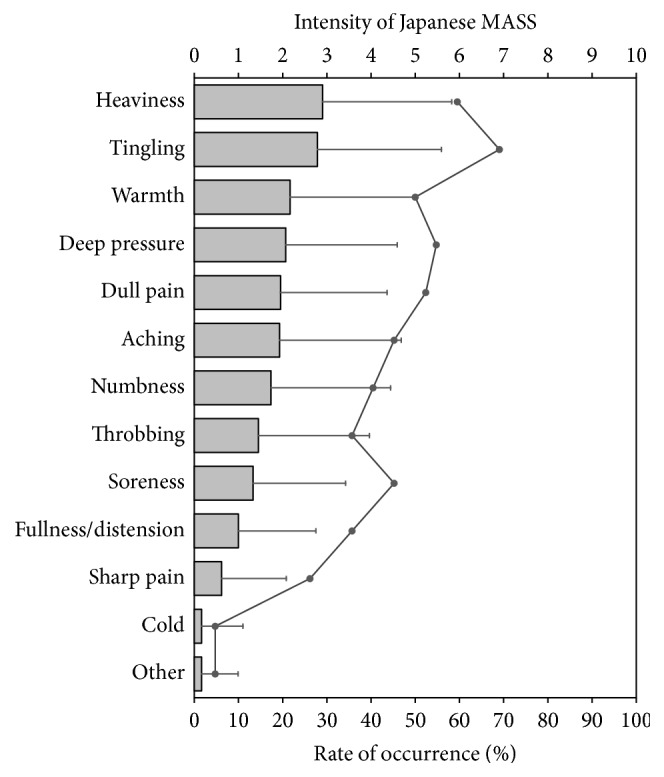
Frequency and intensity of acupuncture sensations on the Japanese MASS (*n* = 42). The line graph indicates frequency. The grey bar graph indicates intensity and the error bars represent the standard deviation.

For the 42 subjects, the mean ± SD intensity for the total MASS score was 20.3 ± 18.3. The mean ± SD of MASS indices calculated from the scores for the 12 descriptors was 5.3 ± 3.8. The largest mean sensation intensity among the 12 descriptors was for “heaviness” and the second largest was for “tingling.” The smallest mean sensation intensity was for “cold” and the second smallest was for “sharp pain” (Figure 3).

In terms of the frequency of occurrence, “tingling” was felt by 70% of subjects, showing the highest occurrence of the 12 descriptors. Less than 5% of subjects felt “cold.” The frequencies and intensities of the sensations for the 12 descriptors were positively correlated; that is, the more frequent the occurrence, the higher the intensity (Figure 3). Subjects reported few sensations other than the 12 descriptors. The mean ± SD occurrence of sensations in the descriptors reported by a single subject was 5.1 ± 3.1.

There were no significant differences between males and females, or between right and left LI4 for the S-VAS scores, total MASS scores, and MASS indices.

### 3.4. Internal Consistency

Overall, Cronbach's alpha coefficient for the 12 descriptors was 0.860. The largest value for 11 descriptors was 0.865 with the exclusion of “cold,” the second largest was 0.856 with the exclusion of “soreness,” and the smallest was 0.835 with the exclusion of “warmth.” The corrected item-total correlation for “cold” was 0.154, while the others ranged from 0.427 to 0.734.

### 3.5. Criterion-Related Validity

Total MASS scores and MASS indices correlated significantly with total SF-MPQ scores, S-VAS scores, and SF-MPQ sensory dimension scores. The Pearson's correlation coefficient between total MASS scores and SF-MPQ affective dimension scores was larger than that between MASS indices and affective dimension scores (Table 1).

### 3.6. Construct Validity (Factor Analysis)

The factor analysis using a scree plot criterion loaded the 12 descriptors onto two factors. The cumulative contribution ratio was 0.561. The Kaiser-Meyer-Olkin statistic, which indicates sampling adequacy, was 0.796, and Bartlett's test of sphericity was *χ*^2^(66) = 201.32  (*p* < 0.001). Factors 1 and 2 were classified as “varying pain sensation” and “pressure sensation depth under the skin” (Table 2).

## 4. Discussion

The original English MASS was translated into Japanese to develop a reliable and valid Japanese MASS for use in evaluating acupuncture sensations, or de qi, in acupuncture trials with Japanese populations. The results obtained from our reliability and validation tests showed that the Japanese MASS is a reliable and valid questionnaire to evaluate acupuncture sensations associated with needle insertion or manipulation in a Japanese sample. Our study yielded the first reliable and valid Japanese version of the MASS.

### 4.1. Translation into Japanese

In the Oxford English Dictionary, “soreness” is defined as “a raw or painful place on the body.” Translating “soreness” into “feeling of languid” in Japanese might have been biased due to the translator's previous experience with acupuncture. However, this biased translation was highlighted in the backtranslation and amended by the expert committee. There was no discrepancy between the two backtranslators in the backtranslation process, even though one backtranslator had a medical/acupuncture background, which is not consistent with Beaton's guidelines. This suggests that the backtranslation process avoided information bias and elicited no unexpected meanings for the descriptors. Given the amendment of the initial forward translation of “soreness” in the third and fourth stages of our cross-cultural adaptation process, we believe that the meanings of the descriptors in the Japanese MASS are equivalent to those in the English MASS.

### 4.2. Content Validity

A relatively large proportion of acupuncturists in our study disagreed with “cold” being an acupuncture sensation, which might be because patients and subjects rarely reported a “cold” sensation during needling. In the original and Chinese MASS study, “cold” was included because coldness and warmth are two of the earliest sensations to be symptom-specific based on the vacuity-repletion theory [32] in ancient literature [2, 22], which is core concept in acupuncture treatment. Therefore, although the frequency and intensity of “cold” sensations felt by subjects during needling were distinctively smaller when compared with the other descriptors, as well as in previous studies [2, 18, 25], we included “cold” to be consistent with original version of the MASS.

The CVR for “fullness/distension” was lower than that for “cold.” However, we found the average intensity and frequency of “fullness/distension” were higher than those of “cold”; therefore, we kept the descriptor in the MASS.

### 4.3. Internal Consistency

The overall Cronbach's alpha coefficient for the descriptors (0.860) was between 0.70 and 0.95, indicating that the Japanese MASS had good internal consistency [33]. This was further supported by the fact that there were almost no changes in the Cronbach's alpha coefficients when individual descriptors were deleted in turn. The alpha coefficient exceeded 0.860 only when “cold” was deleted, and the extremely low corrected item-total correlation for “cold” indicated that “cold” was a heterogeneous descriptor. However, we left “cold” in place to compare with the other versions of the MASS.

### 4.4. Criterion-Related Validity

The criterion-related validity of the Japanese MASS was demonstrated by strong correlations between total MASS scores and total SF-MPQ scores and between total MASS scores and the SF-MPQ sensory dimension scores. These strong correlations were expected, as four descriptors classified as sensory from the SF-MPQ are included in the MASS, and the descriptors in the ASS are closely related to those in the MPQ [21]. Acupuncture sensations involve a pain component and it could be said that the MASS is another form of a questionnaire that explores various aspects of pain sensation, especially deep, dull pain.

The SF-MPQ affective dimension scores were moderately correlated with the total Japanese MASS scores, whereas no correlation was found with the Chinese MASS [25]. Approximately 70% of Chinese acupuncturists reported that their patients liked de qi. In contrast, most Japanese patients seem to not like the acupuncture sensations, similar to many people in the US [34]. Japanese people may recognise de qi as an uncomfortable sensation and may also perceive it negatively.

The stronger correlation between the S-VAS scores and the MASS indices than between the S-VAS scores and total MASS scores indicates that the Japanese MASS indices expressed the total amount of acupuncture sensations well. Therefore, the Japanese MASS works as well as the original MASS in studying acupuncture sensations.

### 4.5. Construct Validity (Factor Analysis)

Although the 12 descriptors were classified into five factors in the Chinese MASS validation study, they were classified into two meaningful factors in the Japanese MASS, which corresponded to the two factors in the original MASS [2]. This indicates that the Japanese MASS retained the same factor structure as the original MASS.

The Japanese MASS presented in this study will allow Japanese acupuncture researchers to investigate acupuncture sensations in clinical studies with Japanese populations. In order to understand acupuncture sensations in terms of clinical efficacy from an ethnical perspective, further exploration is necessary to compare the de qi profiles in the Japanese MASS with those obtained by the original MASS and the Chinese MASS.

## 5. Conclusion

Our study demonstrated that the Japanese MASS has good reliability, content validity, criterion-related validity, and construct validity. Therefore, the Japanese MASS is a valid and reliable instrument for use with Japanese populations.

## Figures and Tables

**Table 1:** Pearson's correlation coefficients between total MASS scores and MASS indices versus SF-MPQ total scores, sensory dimension scores, affective dimension scores, and S-VAS scores (*n* = 42).

	Total SF-MPQ scores	SF-MPQ sensory dimension scores	SF-MPQ affective dimension scores	S-VAS scores
Total MASS scores	0.777^*∗∗∗*^	0.804^*∗∗∗*^	0.609^*∗∗∗*^	0.691^*∗∗∗*^
MASS indices	0.673^*∗∗∗*^	0.603^*∗∗∗*^	0.364^*∗*^	0.803^*∗∗∗*^

^*∗∗∗*^ *p* < 0.001; ^*∗*^*p* < 0.05.

**Table 2:** Factor loadings of the Japanese MASS: two factors (*n* = 42).

Descriptors	Factors	
	Varying pain sensation	Pressure sensation depth under the skin
Throbbing	0.766	
Numbness	0.722	
Aching	0.668	
Cold	0.648	
Sharp pain	0.632	
Tingling	0.596	
Dull pain	0.555	0.451
Heaviness		0.793
Deep pressure		0.764
Soreness		0.723
Warmth	0.474	0.651
Fullness/distension		0.612

Note: factor loadings over 0.45 are exclusively represented.
